# TRAIL receptor gene editing unveils TRAIL-R1 as a master player of apoptosis induced by TRAIL and ER stress

**DOI:** 10.18632/oncotarget.14285

**Published:** 2016-12-27

**Authors:** Florent Dufour, Thibault Rattier, Andrei Alexandru Constantinescu, Luciana Zischler, Aymeric Morlé, Hazem Ben Mabrouk, Etienne Humblin, Guillaume Jacquemin, Eva Szegezdi, Fabien Delacote, Naziha Marrakchi, Gilles Guichard, Catherine Pellat-Deceunynck, Pierre Vacher, Patrick Legembre, Carmen Garrido, Olivier Micheau

**Affiliations:** ^1^ INSERM, UMR866, « Equipe labellisée Ligue contre le Cancer » and Laboratoire d’Excellence LipSTIC, Dijon, France; ^2^ Univ. Bourgogne Franche-Comté, Dijon, France; ^3^ Pós-graduação emCiências da Saúde, Escola de Medicina, Pontifícia Univ. Católica do Paraná, Curitiba, Paraná, Brazil; ^4^ Laboratoire des Venins et Biomolécules Thérapeutiques LR11IPT08, Institut Pasteur de Tunis, Tunis, Tunisia; ^5^ Department of Biochemistry and National Centre for Biomedical Engineering Science, National University of Ireland, Galway, Ireland; ^6^ Cellectis, Paris, France; ^7^ Univ. de Bordeaux, CNRS, IPB, UMR 5248, CBMN, Institut Européen de Chimie et de Biologie, Pessac, France; ^8^ INSERM, UMR892, CNRS 6299, Université de Nantes, Nantes, France; ^9^ INSERM U1218, Univ. de Bordeaux, Institut Bergonié, Bordeaux, France; ^10^ CLCC Eugène Marquis, INSERM ER440 Oncogenesis, Stress & Signaling, Rennes, France; ^11^ Centre Georges-François Leclerc, Dijon, France

**Keywords:** receptor, TRAIL, signaling, apoptosis, cancer

## Abstract

TRAIL induces selective tumor cell death through TRAIL-R1 and TRAIL-R2. Despite the fact that these receptors share high structural homologies, induction of apoptosis upon ER stress, cell autonomous motility and invasion have solely been described to occur through TRAIL-R2. Using the TALEN gene-editing approach, we show that TRAIL-R1 can also induce apoptosis during unresolved unfolded protein response (UPR). Likewise, TRAIL-R1 was found to co-immunoprecipitate with FADD and caspase-8 during ER stress. Its deficiency conferred resistance to apoptosis induced by thaspigargin, tunicamycin or brefeldin A. Our data also demonstrate that tumor cell motility and invasion-induced by TRAIL-R2 is not cell autonomous but induced in a TRAIL-dependant manner. TRAIL-R1, on the other hand, is unable to trigger cell migration owing to its inability to induce an increase in calcium flux. Importantly, all the isogenic cell lines generated in this study revealed that apoptosis induced TRAIL is preferentially induced by TRAIL-R1. Taken together, our results provide novel insights into the physiological functions of TRAIL-R1 and TRAIL-R2 and suggest that targeting TRAIL-R1 for anticancer therapy is likely to be more appropriate owing to its lack of pro-motile signaling capability.

## INTRODUCTION

TRAIL/APO2L selectively triggers cell death in a large variety of human tumors [1]. This promising anti-tumor protein belongs to the TNF subfamily of apoptosis-inducing ligands [2] and has attracted much interest in oncology. However, the use of TRAIL or its derivatives in the clinic has, so far, failed to demonstrate its efficacy, despite clear evidence of antitumor capabilities [3]. Apoptosis triggered by TRAIL occurs through its binding to TRAIL-R1 and/or TRAIL-R2, also coined DR4 and DR5, respectively [4]. Activation of these cell surface receptors by TRAIL induces the formation of a so-called death-inducing signaling complex (DISC) on the cytosolic tail of the receptor, where the apoptosis-initiating caspase-8 and caspase-10 proteins are brought in close proximity by the adaptor protein FADD [5]. Recruitment of these two initiator caspases, through homotypic protein/protein interactions within the TRAIL DISC, allows their activation and the subsequent release of their active cleaved forms to the cytosol where they activate by proteolytic cleavage effector caspases such as caspase-3, driving cell dismantling.

Both TRAIL agonist receptors share high structural homology and the ability to induce apoptosis through the formation of a DISC. Despite similar expression levels of agonist receptors in a given cancer cell type, engagement of apoptosis can occur either through TRAIL-R1 or TRAIL-R2 [6, 7]. The reasons for this selective engagement remain unclear, so far. In addition, recent evidence suggests that TRAIL-R2, may also display pro-tumorigenic potential [8, 9] and contribute to endoplasmic reticulum stress-induced cell death in a ligand-independent manner [10]. With the aim of assessing selective signaling capabilities of TRAIL-R1 over TRAIL-R2, we generated TRAIL receptor deficient cells using a TALEN approach. The generated isogenic cell lines deficient for TRAIL-R1 and/or TRAIL-R2 allowed us to demonstrate that TRAIL-R1 and TRAIL-R2 display both similar and divergent signaling capacities. Our findings demonstrate that TRAIL-R1 is also able to contribute to cell death induced by unresolved unfolded-protein-response signals and that only TRAIL-R2 is able to induce a calcium-dependent cell motility signaling activity. Importantly, although both receptors are able to induce apoptosis upon TRAIL binding, TRAIL-R1 appears to be more critically required than TRAIL-R2 for apoptosis triggering.

## RESULTS

### TRAIL-induced apoptosis proceeds preferentially through TRAIL-R1

**Figure 1:**
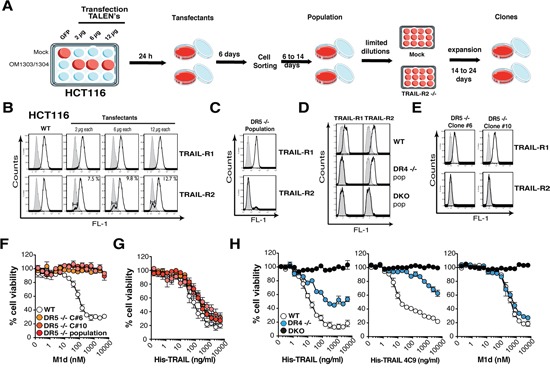
Generation of TRAIL-Receptor-deficient HCT116 cells using the TALEN technology **A**. Schematic scheme of the protocol performed to generate TRAIL-R1- or TRAIL-R2-deficient HCT116 tumor cells. Cells seeded in 12 well plates were transfected with increasing amounts of TRAIL-R2 TALEN plasmid pairs (e.g. OM1303 and OM1304 for TRAIL-R2). After 24 hours transfectants were amplified in a 10 cm dish, cultured for 6 days, then sorted by flow cytometry for their lack of TRAIL-R1 or TRAIL-R2 expression and amplified for a further 6 to 14 days before second cell sorting (population). TRAIL-R2-deficient clones were obtained by limiting dilution. **B-E**. Parental (WT), transfectants, TRAIL-R1 and/or TRAIL-R2-deficient populations and clones (DR4-/-, DR5 -/- or DKO) were assessed by flow cytometry for TRAIL-R1 or TRAIL-R2 expression as indicated. **F-G**. Cell viability of parental (WT), DR5 -/- population or DR5 -/- clones (C#5 and C#10) to increasing concentrations of (F) TRAIL-R2 selective peptidomimetic (M1d) and (G) recombinant TRAIL (His-TRAIL). **H**. Cell viability of parental (WT), TRAIL-R1-deficient (DR4 -/-) or TRAIL-R1 and TRAIL-R2-deficient HCT116 cells (DKO) to increasing concentrations of recombinant TRAIL (His-TRAIL), TRAIL-R1 selective recombinant TRAIL (4C9) or TRAIL-R2 selective peptidomimetic (M1d) assessed by methylene blue as above.

TRAIL signal transduction pathways are fairly well understood, but the specific contribution of TRAIL-R1 and TRAIL-R2 in implementing apoptotic or non-apoptotic signaling remains still poorly understood. In order to get a better understanding of TRAIL-R1 and TRAIL-R2 specific signaling capabilities, we generated TRAIL receptor-deficient isogenic cell derivatives of the colorectal carcinoma cell line HCT116 using a Transcription activator-like effector nucleases (TALEN) gene editing approach. TRAIL-R1 and TRAIL-R2 right and left TALE were designed to target exon 1 and exon 3, respectively (Supplementary Figure 1A). TRAIL-R1- and/or TRAIL-R2-deficient cells were obtained after transfection and successive sorting of cells lacking TRAIL receptor expression by flow cytometry (Figure 1A-1D). TRAIL-R2-deficient (DR5 -/-) clones were obtained by limiting dilution and expansion for 14 to 24 days (Figure 1E). Genomic alterations were confirmed by deep sequencing. As expected, and contrary to parental cells, all HCT116 isogenic TRAIL-R1-deficient (DR4-/-) and TRAIL-R1/TRAIL-R2 double knockout (DKO) cell populations (Pop) as well as the selected TRAIL-R2-deficient (DR5-/-) clone displayed extensive insertions, deletions or substitutions events in the corresponding targeted locus (Supplementary Figure 1B). Compared to the parental cells, all DR5 -/- isogenic cells (population or derived clones) displayed complete resistance to cell death induced by the TRAIL-R2 selective peptidomimetic M1d (Figure 1F), but only moderate protection to His-TRAIL (Figure 1G). On the other hand, loss of TRAIL-R1 caused a pronounced protection against His-TRAIL-induced cell death, and as expected deletion of both receptors fully protected HCT116 cells (DKO) against both TRAIL and TRAIL derivatives (Figure 1H). DR4-/- HCT116 cells displayed, a residual sensitivity to high concentrations of the TRAIL-R1 selective ligand 4C9, albeit to a much lower extent than WT His-TRAIL (Figure 1H). 4C9 is a mutated version of TRAIL, selected for its higher affinity for TRAIL-R1 and low affinity for TRAIL-R2 [11]. Since, HCT116 DKO cells display complete resistance to 4C9, the residual activity of 4C9 in DR4-/- HCT116 cells was attributed to its ability to engage apoptosis through TRAIL-R2 at a high concentration.

**Figure 2:**
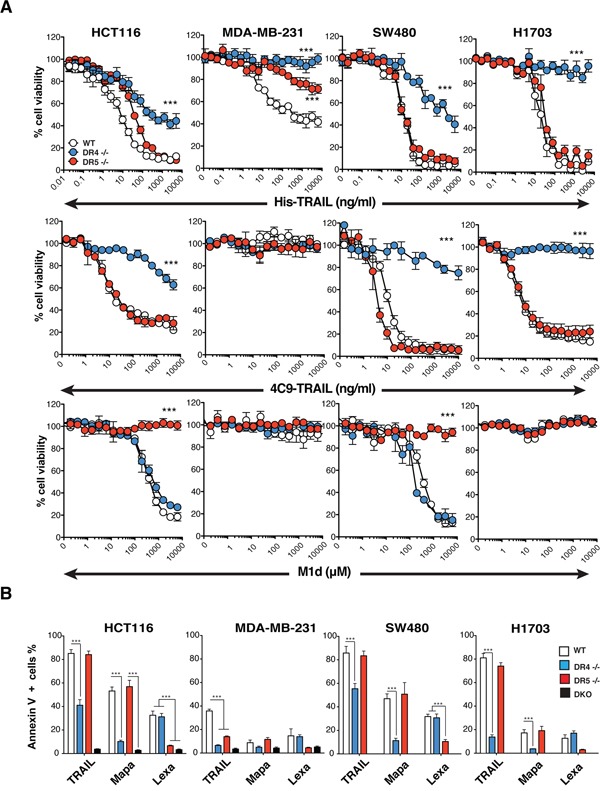
TRAIL-induced apoptosis is preferentially triggered by TRAIL-R1 **A**. Cell viability measured by methylene blue in HCT116, MDA-MB231, SW480 and H1703 parental cells (WT, white circles) or corresponding isogenic TRAIL-R1 (DR4 -/-, blue circles) or TRAIL-R2 (DR5 -/-, red circles) deficient cells exposed to increasing concentrations of His-TRAIL or 4C9-TRAIL and M1d. Data represents mean ± SD of three independent experiments. **B**. HCT116, MDA-MB231, SW480 and H1703 parental cells and their TRAIL-deficient isogenic derivatives were stimulated with 1 μg/ml His-TRAIL or 5 μg/ml Mapatumumab (Mapa) or Lexatumumab (Lexa) for 8 h and analysed for apoptosis with annexinV staining by flow cytometry. Data represent the mean ± SD of at least three different experiments. (***P<0.05 respective to parental cells, one-way ANOVA).

**Figure 3:**
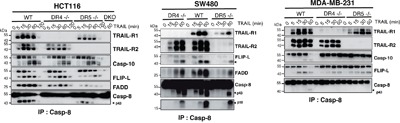
Loss of TRAIL-R1 impairs caspase-8 activation within TRAIL DISC **A**. TRAIL DISC formation was analysed in HCT116, MDA-MB231 and SW480 parental or corresponding TRAIL receptor-deficient cells after stimulation with His-TRAIL for the indicated period of time on intact cells and immunoprecipitation using an anti-caspase-8 antibody followed by immunoblotting with indicated antibodies. Black arrows show cleaved caspase-8 products (p43 and p18).

The substantial reduction of TRAIL-sensitivity in the DR4-/- HCT116 cells is not due to damaged TRAIL-R2 functionality, as evidenced by their fully retained sensitivity to M1d (Figure 1H). To confirm that the HCT116 isogenic cell clones or populations generated are strictly interfering with TRAIL signaling, cells were stimulated with increasing concentrations of Fas ligand. Methylene blue staining, demonstrated that Fas ligand-induced cell death remained unaltered in all these isogenic cells, irrespective of the concentration (Supplementary Figure 1C). In order to substantiate these findings, additional TRAIL-receptor-deficient isogenic cell lines were generated generated, from MDA-MB-231 breast cancer cells, SW480 colorectal carcinoma cells and H1703 lung cancer cells (Supplementary Figure 2A). Irrespective of the cell type, deletion of TRAIL-R1 in these cells (DR4-/-) was always more detrimental to TRAIL-induced cell death than deletion of TRAIL-R2 (DR5-/-) (Figures 2A and Supplementary Figure 2B). Remarkably, SW480 and H1703 DR5-/- cells were almost as sensitive as parental WT cells to TRAIL (Figure 2A). As expected, DR5-/- cells were equally sensitive to the TRAIL-R1 selective TRAIL ligand 4C9 as the parental cells, whereas DR4-/- cells displayed high resistance to 4C9 (Figure 2A). Like HCT116 DR5-/- cells, SW480 cells deficient for TRAIL-R2 were also protected against M1d-induced cell death (Figure 2A). Parental H1703 and MDA-MB-231 and TRAIL receptor-deficient isogenic cells on the other hand displayed complete resistance to M1d (Figure 2A). In agreement with these results, DR4-/- isogenic cells displayed much higher resistance to TRAIL-induced apoptosis than DR5-/- cells, as monitored by annexin V staining (Figure 2B). As expected, DR4-/- and DR5-/- cells were also protected against apoptosis induced by Mapatumumab and Lexatumumab, two agonist antibodies selectively targeting TRAIL-R1 and TRAIL-R2, respectively (Figure 2B). Although, TRAIL DISC composition was not significantly different in cells expressing either TRAIL-R1 or TRAIL-R2, as compared to parental cells (Figure 3), activation of the caspase-8, as evidenced by its cleaved products, was severely impaired in cells lacking TRAIL-R1 (DR4-/-). These findings altogether indicate that TRAIL-induced apoptosis in these cells is mainly triggered by TRAIL-R1.

### TRAIL-induced cell motility is solely mediated through TRAIL-R2

**Figure 4:**
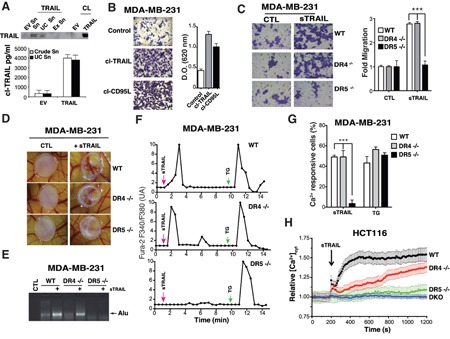
TRAIL-R2, but not TRAIL-R1 induces TRAIL-dependent pro-motile signalling **A**. CHO cells were transfected with an expression vector encoding TRAIL or an empty vector (EV) then cell lysates (CL), culture supernatant (Sn), ultracentrifugated culture supernatant (UC Sn) and exosomes (Ex Sn) were analysed for TRAIL expression by immunobloting. Lower panel, soluble TRAIL production (cl-TRAIL) from crude supernatant or supernatant obtained after ultracentrifugation was measured by ELISA. **B**. MDA-MB-231 cells were serum-starved overnight, seeded in the presence of low serum (0.5%) with or without cl-TRAIL or cl-CD95L (100 ng/ml) for 24 h in a Boyden chamber and migration was assessed by staining with Giemsa. A representative image is shown. Right panel: Giemsa-stained cells that migrated to the lower side of the membrane were lysed and absorbance was measured at a wavelength of 620 nm. **C**. The experiment described above was performed using parental and TRAIL-R1 (DR4-/-) or TRAIL-R2 (DR5-/-) deficient MDA-MB-231 cells in the presence or absence of 100 ng/ml Flag-TRAIL (sTRAIL). Right panel: quantification of the migration as fold difference as compared to parental non-stimulated cells. **D**. TRAIL-R2-dependent TRAIL mediated pro-metastatic properties were assessed in chicken embryos (CAM assay) implanted with MDA-MB231 parental or TRAIL receptor-deficient cells stimulated or not with sTRAIL. **E**. Corresponding qualitative PCR analysis of human Alu sequences found in chicken embryo tissues obtained after stimulation with sTRAIL as above. **F**. Representative time course calcium fluxes in parental, DR4 -/- or DR5 -/- MDA-MB231 cells loaded with Fura-2 after stimulation with 100 ng/ml Flag-TRAIL (sTRAIL) or 2 μM thapsigargin (TG). **G**. Quantification of the Ca2+ responsive cells (%) after sTRAIL or TG stimulation. **H**. [Ca^2+^]_CYT_ was assessed in FuraPE3-AM (1 μM)-loaded cells. Ratio values (R=F340/F380) were normalized to pre-stimulated values (R0). Data represent means ± the SD of 3 independent experiments (> 60 cells). Shown are time course of calcium responses to 100 ng/ml Flag-TRAIL (sTRAIL) in parental, DR4 -/-, DR5 -/- or DKO HCT116 cells.

Like CD95L/FasL [12], TRAIL has been reported in some circumstances to induce cell motility, invasion and metastasis [8, 9, 13]. Although non-apoptotic signalling associated with some of these events was assigned to TRAIL-R2 in cancer cells, in a cell autonomous manner, pro-migratory signalling events induced by TRAIL in normal cells remains unknown, except for monocytes for which chemotaxis and migration has been ascribed to TRAIL-R1 [14]. We therefore took advantage of the TRAIL-receptor-deficient isogenic cell lines to determine whether cell motility induced by TRAIL requires TRAIL-R1, TRAIL-R2 or both receptors. In order to address this question and to avoid apoptosis-triggering, we used trimers of TRAIL, which are unable to induce apoptosis (Supplementary Figure 3A). Soluble cleaved TRAIL (cl-TRAIL) was purified from supernatants of CHO cells transiently transfected with full-length TRAIL (Figure 4A), or from bacterial extracts (sTRAIL) [15]. After ultracentrifugation of the conditioned media to remove exosomes, the amount of cl-TRAIL recovered was estimated by ELISA, and used in the Boyden-chamber assay with MDA-MB-231 cells to assess its ability to trigger cell motility. Like cleaved CD95L/Fas Ligand, cl-TRAIL was able to increase MDA-MB-231 cell motility (Figure 4B). Motility induced by cl-TRAIL in these cells was also recapitulated using the purified Flag-tagged TRAIL (sTRAIL) (Figure 4C), a soluble trimer devoid of pro-apoptotic activity in the absence of cross-linking (Supplementary Figure 3A and [16]). Interestingly, deficiency in TRAIL-R2, but not TRAIL-R1, abolished sTRAIL-induced MDA-MB-231 cell motility (Figure 4C). Similar results were obtained in TRAIL-R2-deficient HCT116 isogenic cells with both DR5-/- and DKO cells displaying reduced cell motility after sTRAIL stimulation as compared to WT or DR4-/- (Supplementary Figure 3B). Consistent with this observation, sTRAIL-induced MDA-MB-231 cell invasion *in vivo* involved TRAIL-R2, as assessed using a chick embryo chorioallantoic membrane CAM assay (Figure 4D-4E). Deficiency in TRAIL-R2 inhibited MDA-MB-231 cell migration (Figure 4D) and invasion as demonstrated by the absence of Alu sequences in the chick embryo (Figure 4E). Contrary to DR5-/- cells, WT and DR4-/- cells, which express TRAIL-R2, were able to migrate and invade the host organism (Figure 4D-4E). Cell motility has been reported to be tightly associated with changes in calcium flux [17]. Accordingly, sTRAIL induced a calcium response in WT and DR4-/- MDA-MB-231 cells but not in DR5-/- (Figure 4F-4G) or DKO MDA-MB-231 cells (Supplementary Figure 3C). Notwithstanding, all MDA-MB-231 parental or isogenic cells were able to respond to thapsigargin, a non-competitive sarco/endoplasmic reticulum Ca2+ pumps (SERCAs) inhibitor (Figure 4F-4G), indicating that the calcium response is selectively induced by TRAIL-R2 upon sTRAIL stimulation. These results were confirmed in HCT116 cells (Figures 4H and Supplementary Figure 3D). It should be noted here that migration induced by TRAIL-R2 is non-self autonomous, as spontaneous migration of HCT116 and MDA-MB-231 cells is not altered in the absence of the receptor, as compared to parental or DR4 -/- cells (Supplementary Figure 3E). However, migration induced by FCS was clearly reduced in TRAIL-R2-deficient cells, suggesting that soluble TRAIL may be present in FCS, in addition to other chemoattractants (Supplementary Figure 3E).

### Unresolved UPR-mediated apoptosis involves both TRAIL-R2 and TRAIL-R1

**Figure 5:**
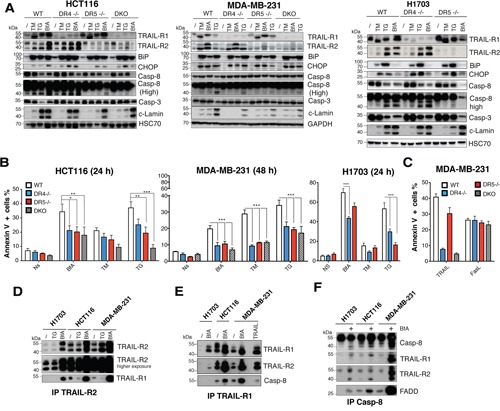
TRAIL-R1 contributes to ER stress-mediated apoptosis **A**. ER stress was induced in parental HCT116, MDA-MB231 and H1703 cells and their corresponding isogenic TRAIL receptor-deficient variants with 1 μg/ml brefeldin A (BfA), 1 μg/ml tunicamycin (TM) or 100 nM thapsigargin (TG) for the indicated periods of time. Induction of the ER stress markers BiP and CHOP and of apoptosis by activation of pro-caspase-8, -3 and cleavage of lamin A (c-lamin) were detected by immunoblotting. **B**. Cells treated as in (A) were analysed for induction of cell death with annexinV staining and flow cytometry. Data represent the mean ± SD of at least three different experiments. (***P<0.05 respective to parental cells, one-way ANOVA). **C**. Indicated MDA-MB231 isogenic cell lines were stimulated with 1 μg/ml His-TRAIL (TRAIL) or 1 μg/ml Fc-FasL (FasL) and apoptosis was analyzed as above. **D-F**. Parental HCT116, H1703 or MDA-MB-231 cells were stimulated with 1 μg/ml brefeldin A (BfA) or 100 nM thapsigargin (TG) for 24 or 48 h as in (A) and cell extracts were subjected to immunoprecipitation using either an (D) anti-TRAIL-R2, (E) anti-TRAIL-R1 or (F) anti-caspase-8 antibody before analysis by immunoblot with indicated antibodies.

TRAIL-R2 is known to be regulated and contribute to ER stress-mediated apoptosis, independent of its cognate ligand [10]. Accordingly, in the three cell lines tested namely HCT116, H1703 and MDA-MB-231, thapsigargin (TG), tunicamycin (TM) and brefeldin A (BfA) induce up-regulation of TRAIL-R2 in parental or DR4 -/- cells, but not in DR5-/- and DKO isogenic cells (Figure 5A), and in agreement with previous findings [10], deficiency in TRAIL-R2 significantly protected HCT116 cells from apoptosis induced by TG (Figures 5B and 4A). As expected, HCT116 DKO cells were also significantly less sensitive than parental cells to TG-induced apoptosis. Protection against TG-induced apoptosis mediated by TRAIL-R2 deficiency was also evidenced in H1703 and MDA-MB-231 cells (Figures 5B and Supplementary Figure 4A-4B). Remarkably, loss of TRAIL-R1 expression also afforded significant protection against TG-induce apoptosis in H1703 and MDA-MB-231 cells and less so in HCT116 cells (Figure 5B), indicating that TRAIL-R1 contributed equally to ER stress-mediated apoptosis. Moreover, both TRAIL-R1- and TRAIL-R2-deficient cells were significantly protected against BfA-induced apoptosis in HCT116 and MDA-MB-231 cells, as well as in H1703 cells, albeit to a lower extent for the DR5-/- isogenic derivative (Figures 5B and 4A-4B). Protection against TM-induced apoptosis was, on the other hand, only evidenced in the most sensitive cell line, MDA-MB231 (Figures 5B and Supplementary Figure 4A-4B). Resistance to ER-stress inducers, as shown here with MDA-MB-231 isogenic cells, was not associated with a downstream disability to transduce apoptosis, since apoptosis-induced by Fas ligand, another ligand of the TNF superfamily, was unaffected whether the cells harbored or not these receptors (Supplementary Figure 4C).

Interestingly, although BIP or CHOP were induced after ER stress stimulation in all cell lines, and despite the fact that they likely contribute to both TRAIL-R1 and TRAIL-R2 up-regulation [10, 18] (Figure 5A), the percentage of cells undergoing apoptosis varied both depending on the cell line and the ER stress-inducer (Figure 5A). These finding suggest that UPR-mediated apoptosis may not be strictly correlated with transcriptional regulation of TRAIL-R1 and TRAIL-R2. Accordingly, regulation of TRAIL-R1 and TRAIL-R2 expression levels in WT HCT116 and MDA-MB-231 cells did not correlate with apoptosis induced by these compounds. For instance while BfA induced stronger up-regulation of both receptors as compared to TG in both cell lines (Figure 5A), it induced weaker apoptosis than TG or TM in MDA-MB-231 cells (Figure 5B). Irrespective of whether apoptosis-induced by TRAIL receptors upon ER stress is directly linked to their transcriptional up-regulation, and in agreement with previous findings [10], apoptosis involved formation of a complex associating caspase-8, FADD, TRAIL-R1 and TRAIL-R2 in a TRAIL-independent manner (Figure 5D-5F). Likewise, stimulation of parental HCT116 and MDA-MB-231 cells with TG or BfA induced the formation of heteromers of TRAIL-R1 and TRAIL-R2, as evidenced by immunoprecipitation (Figure 5D-5E) and the formation of a DISC-like complex [10] (Figure 5F). Our results therefore support the finding that both TRAIL-R1 and TRAIL-R2 are able to form an active DISC-Like complex upon BfA stimulation and contribute, at least in part, to apoptosis induced by ER stress inducers.

## DISCUSSION

TRAIL and its agonist receptors have been considered for anticancer therapies for over two decades [3]. TRAIL-R1 and TRAIL-R2 display 50 to 53% overall homology and share the ability to recruit FADD and caspase-8, allowing apoptosis triggering. Although TRAIL binding affinity for TRAIL-R2 was shown to be slightly stronger than TRAIL-R1 [11, 19], apoptosis is preferentially engaged through TRAIL-R1 in chronic leukemic lymphocytic cells, despite high expression levels of TRAIL-R2 [7, 20, 21]. While both receptors are often co-expressed in tumor cell lines, and albeit loss of TRAIL-R1 can be found in many tumor cell lines, occurrence of a TRAIL-R2-deficiency in TRAIL-R1 expressing cells is rare, and has to our knowledge only been described in the human erythroleukemia cell line Hel [22]. So far, the studies aiming at analyzing the specific contribution of TRAIL-R1 or TRAIL-R2 during TRAIL-induced apoptosis have mostly relied on mutated TRAIL ligands [11], peptidomimetics [23] or agonistic monoclonal antibodies [24]. However, these agonists introduce bias, due either to their residual affinity for non-targeted receptors or to their reduced potency as compared to TRAIL itself.

In this work we have generated TRAIL-receptor-deficient isogenic cell lines to analyze the relative contribution of each TRAIL agonist receptor in triggering apoptosis or non-apoptotic signaling using the same ligand. Our findings, that apoptosis induced by TRAIL in a small panel of carcinoma cell lines is preferentially induced by TRAIL-R1 together with the demonstration that TRAIL-induced migration is solely triggered by TRAIL-R2 or that TRAIL-R1 is able to contribute to apoptosis induced by ER stress inducers, uncover TRAIL-R1 as an important target for cancer therapy. Until now, most studies have focused on TRAIL-R2, neglecting TRAIL-R1 [3]. Accordingly, only one anti-TRAIL-R1 antibody (Mapatumumab) has been assessed in clinical trials, whereas 5 distinct anti-TRAIL-R2 antibodies have been tested, so far [3, 25]. TRAIL-R2 has probably attracted more attention than TRAIL-R1 owing to the fact that its expression is more prone to transcriptional regulation by genotoxic or non-genotoxic compounds and that its increase in expression is often associated with increased sensitivity to TRAIL-induced cell death [10, 26, 27]. However, as demonstrated here using TRAIL-deficient isogenic cell lines, and in a recently published work using a siRNA approach [8], TRAIL-R2 but not TRAIL-R1, can induce tumor cell motility and contribute to metastasis *in vivo*. In contrast to the cell autonomous pro-motile signaling capability attributed to TRAIL-R2 [8], our present work indicates that this signaling property requires TRAIL. Along this line, we have shown in the past that even the most potent soluble TRAIL (His-TRAIL) can induce the migration of HCT116 cells exhibiting a deficiency in the intrinsic pathway, due to the absence of Bax expression or to the overexpression of Bcl-2 anti-apoptotic family members [9]. This non-apoptotic signaling is also shared by Fas [12], another member of the TNF superfamily. TRAIL-R2 pro-motile signaling should thus considered as a potential threat for TRAIL-based cancer therapy, as it may potentially lead to increased occurrence of metastasis.

TRAIL-R1, on the other hand is unable to trigger this non-apoptotic signaling pathway, but is extremely potent in triggering TRAIL-induced apoptosis. Moreover, the present work reveals for the first time that, like TRAIL-R2, TRAIL-R1 is also able to contribute to unresolved UPR-mediated apoptosis. In the course of ER stress, TRAIL-R1 is engaged in a macromolecular complex including FADD and caspase-8 and contributes to apoptosis induced by ER stress inducers.

While additional work will be required to fully understand why TRAIL engages apoptosis preferentially through TRAIL-R1, or how TRAIL-R1 engages the caspase-8 machinery in the absence of TRAIL during unresolved UPR, our findings provide novel insights to TRAIL agonist receptors physiological function and delineate TRAIL-R1 as a priority for cancer therapy.

## MATERIALS AND METHODS

### Cell lines

The human colon carcinoma HCT116 and SW480 (ATCC), breast carcinoma MDA-MB-231 (ATCC) were maintained in DMEM (Lonza, Levallois-Perret, France) with 10% fetal calf serum. The lung carcinoma H1703 (kind gift from Pr Lebecque, Lyon, France) was maintained in RPMI (Lonza) as above. Cells were cultured in 5% CO_2_. Chinese hamster ovary (CHO) cells were maintained in DMEM as above, and in OPTI-MEM (Lonza) after transfection.

### Ligand production, chemicals and antibodies

Flag-tagged recombinant soluble human TRAIL, His-tagged human WT-TRAIL or 4C9 (selective for TRAIL-R1) or M1d (selective TRAIL-R2 peptidomimetic) were produced or obtained as described previously [11, 23, 28]. Mapatumumab and Lexatumumab were kind gifts from Human Genome Sciences (Rockville, MA, USA). Brefeldin A, thapsigargin, tunicamycin and the anti-Flag M2 antibody were purchased from Sigma-Aldrich (Lyon, France). For western blot analysis, anti-TRAIL-R1 (Cat# AB16955) and TRAIL-R2 (Cat# AB16942) antibodies were purchased from Chemicon (Millipore, Molsheim, France), anti-FADD (Cat# 610400) from Transduction Laboratories (BD biosciences, Le Pont de Claix, France), antibodies against caspase-3 (clone 8G10), caspase-8 (clone 5F7), caspase-10 (clone 4C1) and cleaved lamin A (Asp230) were all from Medical & Biological Laboratories (Clinisciences, Montrouge, France). Anti-BIP (Cat#3177), CHOP (Cat#2895), PDI (Cat#3501), PERK (Cat#5683), TRAIL-R1 (Cat#8074) were obtained from Cell Signaling Technology (Ozyme, Saint Quentin, France). Anti-FLIP antibody (clone Dave-2) was from Adipogen (Coger, Paris, France), anti-cleaved lamin A/C Asp230 (Cat#3596-1) from Epitomics (Abcam, Paris, France). Anti-GAPDH (clone 0411) and HSC70 (clone B-6) antibodies were from Santa Cruz Biotechnology (CliniSciences, Nantere, France). Anti-TRAIL antibody (MAB687) was from R&D systems (Lille, France). HRP-conjugated anti-rabbit or mouse secondary antibodies were from Jackson ImmunoResearch (Interchim, Montluçon, France) while HRP-conjugated anti-mouse IgG1-, Ig2a- and Ig2b-specific antibodies were from Southern Biotech (Clinisciences). For flow cytometry experiments, the anti-TRAIL-R1 (clone wB-K32), and TRAIL-R2 (clone B-L27) antibodies were kindly provided by Diaclone (Besançon, France). The secondary antibody was an Alexa-488 conjugated-goat anti-mouse antibody from Molecular Probes (Life technologies, Saint Aubin, France). For immunoprecipitation experiments anti-TRAIL-R1 (clone wB-T29) and anti-TRAIL-R2 (clone B-D37) antibodies were from Diaclone, anti-caspase-8 (clone C20, SC-6136) from Santa Cruz Biotechnology and anti-Histidine (clone AD1.1.10) antibody was from AbDserotec (Bio-Rad, Marnes-la-Coquette, France).

### TALEN plasmids and locus

Plasmids pTAL.EF1a-Left and pTAL.EF1a-Right encoding TRAIL-R1 and TRAIL-R2 custom TALEN® were generated by Cellectis (OM1303 and OM1304 for TRAIL-R2 and OM1307 and OM1308 for TRAIL-R1). The RVD sequences for TRAIL-R1 and TRAIL-R2 were TRAIL-R1 OM1307, NG-HD-HD-NG-NN-NN-HD-NI-NN-NG-NN-NI-HD-NG-HD-NG; OM1308, HD-NG-NN-NG-HD-HD-HD-NI-HD-NG-HD-NN-HD-NG-NN-NG TRAIL-R2 OM1303, NI-NN-NI-NN-NI-NG-NG-NN-HD-NI-NG-HD-NG-HD-HD-NG; OM1304 HD-HD-NI-NN-NG-NN-NI-NN-NG-NN-HD-NG-NI-NG-NI-NG. Equal amounts of each pair of TALENs, ranging from 1 to 6 μg, were introduced by transfection into HCT116 cells grown in 12-well plates using Fugene HD (Promega, Charbonnières, France). Transfected cells were cultured for 6 days at 37°C before sorting out the TRAIL-R1- and TRAIL-R2-deficient cells with cell sorting. The sorted cell populations were allowed to grow for 6-14 days and standard limited dilutions in 96-well plates were performed to expand individual clones. Clonal populations were expanded for 14 to 24 days before screening for loss of TRAIL receptor expression by flow cytometry. To confirm gene disruption, genomic DNA was isolated from individual clones or cell populations or cell populations and the targeted locus was analyzed by deep sequencing.

### Deep sequencing

One to 5 million cells were harvested, washed twice in PBS and genomic DNA was extracted using either a genomic DNA lysis buffer (10 mM TRIS, pH8; 0.45% (V/V) NP40; 0.45% (V/V) Tween20; 100 μg/ml proteinase K) or a genomic DNA extraction kit (DNeasy Blood & Tissue Kit from Qiagen), respectively. Lysates were obtained using the genomic DNA lysis buffer after incubation for 10 min at room temperature, followed by 2 h at 55°C and finally for 10 min at 95°C. These lysates were stored at 4°C for up to one week. Genomic extraction using the DNeasy Blood & Tissue Kit was performed according to the manufacturer's instructions. PCR were performed using the primers containing the adaptor sequences needed for deep-sequencing: forward primer 5′-CCA-TCT-CAT-CCC-TGC-GTG-TCT-CCG-ACT-CAG (adaptor sequence)-10N (bar code sequence allowing PCR product identification)-and locus specific sequence-3′; reverse primer 5′-CCT-ATC-CCC-TGT-GTG-CCT-TGG-CAG-TCT-CAG-(reverse adaptor sequence)- and locus specific sequence-3′. DR4 locus specific sequences: forward 5′-CAA-GTG-GCA-AAA-CGA-CTC-CG-3′ and reverse 5′-CCT-CGT-GGT-TCA-ATC-CTC-CC-3′; DR5 locus specific sequences: forward 5′-AGC-TAG-GTA-GAG-GAG-ATT-TCC-C-3′ and reverse 5′-GGT-ATG-ATG-AAG-ACC-AAG-GTG-GA-3′. PCR products were purified using Agencourt AMPure XP kit (Beckman Coulter, Villepinte, France) and sequenced using one-way reads amplicon sequencing method with the 454 sequencing system from (Roche, Meylan, France) according to manufacturer's instructions. Several thousands of sequences were obtained per PCR product and analyzed for the presence of site-specific insertion or deletion events at the TALEN cleavage site using the Cellectis proprietary algorithm. Analysis took into account one base pair insertion or deletion events.

### Chick chorioallantoic membrane (CAM) assay

Chick embryos from 3-days-old eggs were opened and placed in double Petri dishes with water for humidity. After incubation for 7 days at 37.5°C, 5×10^6^ in Matrigel/serum (B&D) MDA-MB-231 cells expressing both receptors (WT), TRAIL-R2 only (DR4 -/-) or TRAIL-R1 onle (DR5-/- cells) were applied on the CAM on a filter paper disk (diameter of 6 mm). Tumour cell migration was analyzed after 7 days of incubation at 37°C. Cell migration was observed using a digital camera at 10 × magnification and by PCR on whole chick embryo using human Alu sequences. Detection of human Alu sequences from CAM studies was performed on whole chick embryo tissue samples. Genomic DNA was isolated from CAM's tissues using DNA Isolation Kit (DNeasy Blood & Tissue Kit, Qiagen) and Alu sequences were amplified using the following primers : Alu-Forward 5′ ACG-CCT-GTA-ATC-CCA-GCA-CTT 3′and Alu-Reverse 5′ TCG-CCC-AGG-CTG-GAG-TGC-A 3′. PCR analysis was performed in a 50 μl PCR reaction containing 1 μg of cDNA, 2 μl dNTPs (2.5 mmol/l each), 1 μl each of the specific primers (20 μM), 5 μl hot start Taq buffer 10X and 0.5 μl hot start Taq DNA polymerase (5 U/μl; Promega) and amplified as follows: 2 min at 95°C followed by 30 cycles of 30 sec at 95°C, 45 sec at 58°C, 45 sec at 72°C; and a final step of 10 min at 72°C. Products were visualized as described above.

### Cell viability and annexin V assays

TRAIL agonists (His-hTRAIL, 4C9 and M1d), or FasL agonist (Fc-FasL) were titrated in 96-well plates using 5×10^4^ cells per well, incubated at 37°C for 16 hours. Plates were incubated at 37°C for 16 hours with 5% CO_2_, and cell viability was assessed using methylene blue staining as described previously [29]. Apoptosis induced by TRAIL and the above-mentioned TRAIL agonists, as well as Mapatumumab and Lexatumumab was analyzed by flow cytometry using the Annexin V-FITC/PI staining kit purchased from Miltenyi Biotec (Miltenyi Biotec, Paris, France) according to the manufacturer's instructions. Stained cells were analyzed with a BD LSR2 flow cytometer (BD Biosciences, Le Pont de Claix, France). The percentage of Annexin V-positive cells was calculated as the number of cells demonstrating Annexin V staining (PI negative or positive) divided by the total number of cells examined. Experiments were repeated at least three times.

### *In vitro* cell motility assays

For *in vitro* motility assays, cells were serum-starved overnight, detached using trypsin and 10^5^ cells were added onto 8 μm pore-sized membrane top Boyden chambers placed in 24-well plates (Millipore, Molsheim, France). Top chambers were filled with low serum (1%)-containing medium, while the bottom chambers were filled as above in the presence or absence of 100 ng/ml of cleaved CD95L (cl-CD95L), TRAIL (cl-TRAIL) or Flag-TRAIL and the plates were cultured for 24 h at 37°C in a 5% CO2, humidified incubator. Cell invasion was quantified as follows: the non-motile cells from the top side of the membrane were removed mechanically using cotton-tipped swabs, and the invading cells from the reverse side of the membrane were fixed with methanol and stained with Giemsa. For each experiment, representative pictures were taken for each insert, then cells were lyzed and absorbance at 560 nm correlating to the amount of Giemsa stain was measured.

### Calcium video imaging in living cells

Single-cell [Ca2+]cyt imaging was performed ratiometrically, using either Fura-2 AM or FuraPE3-AM calcium indicators. Serum-starved cells were loaded with 1 μM Fura-2 AM or 1 μM FuraPE3-AM at room temperature in Hank's Balanced Salt Solution (HBSS) for 30 min. FuraPE3-AM exhibits limited compartmentalization in intracellular stores and is leakage resistant [30]. Cells were rinsed with HBSS and incubated in the absence of the Ca2+ probe for 15 min to complete dye de-esterification. For Fura-2 AM staining, cells were placed under an inverted fluorescence microscope (Axiovert 40 CFL) and stimulated with Flag-TRAIL (100 ng/ml) or thapsigargin (for the indicated period of time). Images were captured at 510 nm by a fast-scan camera. Fura2-AM was alternately excited at 340 and 380 nm, and ratios of the resulting images (excitations at 340 and 380 nm and emission filter at 520 nm) were produced at constant intervals (2s or 5s according to the stimulus). Fura-2 ratio images (Fratio 340/380) were displayed and the Fratio values from the regions of interest (ROIs) drawn on individual cells were monitored over time and analyzed with the Axiovision software. Each experiment was independently repeated 3 times, and for each experimental condition, an average of more than 10 single-cell traces was displayed. For FuraPE3-AM analysis, cells were stimulated as above and fluorescence micrograph images were captured at 510 nm, using an inverted epifluorescence microscope (Olympus IX70) equipped with a 40x UApo/340-1.15W objective (Olympus), and fluorescence micrograph images were captured at 510 nm and 12-bit resolution by a fast-scan camera (CoolSNAP fx Monochrome, Photometrics). To minimize UV light exposure, a 4×4 binning function was used. Fura-PE3 was alternately excited at 340 and 380 nm, and the ratios of the resulting images (emission filter at 520 nm) were produced at constant 10s intervals. ROIs were drawn on selected recorded cells to restrict data collection to specific regions. Fratio reflected the changes in intracellular Ca2+ concentration. Fluorescence of regions of interest was normalized to baseline fluorescence (F/F0). Each experiment was repeated 3 times, and the average of more than 20 single-cell traces was analyzed. Data was processed using OriginPro 7.5 software (Origin Lab).

### Immunoprecipitations

For native DISC analysis, 8×10^7^ cells were stimulated in 1 ml of complete medium with 1 μg of His-hTRAIL ligand for the specified times at 37°C. Cells were then washed with PBS before lysis in NP40 buffer (1 % NP40, 20 mM Tris-HCl pH 7.5, 150 mM NaCl, and 10 % glycerol) containing a protease inhibitor cocktail. Alternatively, adherent cells were stimulated with thapsigargin or brefeldin A for the indicated periods of time in the absence of His-TRAIL. Cells were then detached and collected using trypsin, and lysed in Triton-X100 (1 % Triton-X100, 30 mM Tris-HCl pH 7.5, 150 mM NaCl). Cell lysates were pre-cleared with Sepharose 6B (Sigma-Aldrich) for 1 hour at 4°C. After centrifugation, the TRAIL receptor DISC was immunoprecipitated overnight at 4°C from the cell lysates in the presence of protein G-coated beads (Amersham Biosciences, Les Ullis, France) and antibodies targeting caspase-8 (C20), 6xHis (clone AD1.1.10), TRAIL-R1 (wB-T29) or TRAIL-R2 (B-D37). Beads were washed four times with the corresponding NP40 or Triton-X100 lysis buffer, and the immunoprecipitated complexes were eluted in loading buffer (63 mM Tris-HCl pH 6.8, 2 % SDS, 0.03 % phenol red, 10% glycerol, 100 mM DTT), after a 5 min incubation at 95°C. Samples were then processed for immunoblot analysis.

### Western blotting

Immunoprecipitates or cell lysates (in NP40 lysis buffer) were separated by SDS-PAGE then transferred to nitrocellulose membranes. Nonspecific binding sites were blocked by incubation in PBS containing 0.05% Tween 20 and 5% powdered milk. Immunoblots were incubated with a specific primary antibody, then an HRP-conjugated secondary antibody. Blots were developed using the Advansta WersternBright chemiluminescence substrate according to the manufacturer's protocol (Diagomics, Blagnac, France).

### Statistical analysis

Statistical analyses were performed by one-way or two-way ANOVA using the GraphPad Prism program. Significant differences in cytotoxicity were determined by the Bonferroni's multiple comparison test. P-values *< 0.05, **< 0.01, ***< 0.001 were considered significant. All experiments were performed at least 3 times.

## SUPPLEMENTARY FIGURES


