# Supplementary material for: TRAIL receptor gene editing unveils TRAIL-R1 as a master player of apoptosis induced by TRAIL and ER stress
Source: Oncotarget. 2016 Dec 27;8(6):9974–85. doi: 10.18632/oncotarget.14285 (PMC5354785; doi:10.18632/oncotarget.14285)
Supplement: Supplementary file 1 [file oncotarget-08-9974-s001.pdf]

TRAIL receptor gene editing unveils TRAIL-R1 as a master player of apoptosis induced by TRAIL and ER stress

SUPPLEMENTARY FIGURES

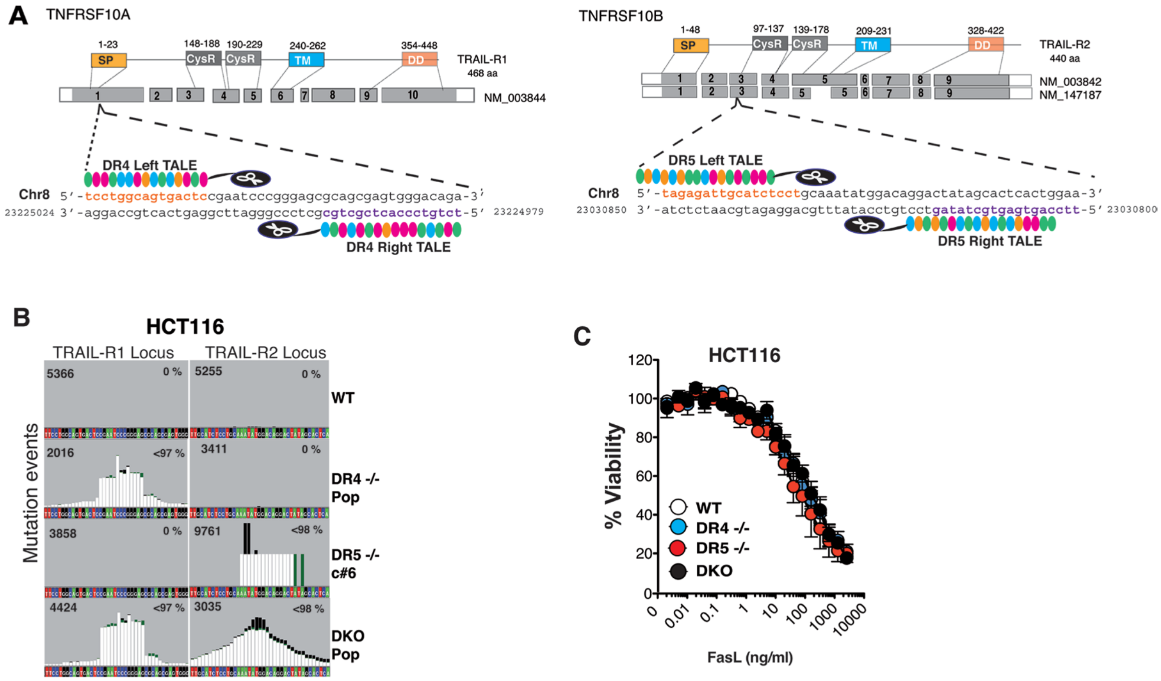

Supplementary Figure 1: TRAIL-R1 and TRAIL-R2 gene editing in HCT116 cells. A. Schematic representation of the loci targeted by custom TRAIL-R1 (DR4) and TRAIL-R2 (DR5) TALENs on chromosome 8 at position 8q21 and 8p22-p21, respectively. Schematic representation of TRAIL-R1 and TRAIL-R2 protein domains and corresponding aa number position are shown above the representative mRNA sequences, displaying corresponding exons. SP stands for signal peptide; cysR for cystein rich repeats, TM for transmembrane domain and DD for Death Domain. Sequences recognized by the left and rights TALENs arms are shown in orange and purple. Positions of the targeted sequences on chromosome 8 are shown on each side of the annotated sequence. **B.** Deep sequencing analysis of mutation events in parental, TRAIL-R1-deficient (DR4-/-), TRAIL-R2-deficient (DR5-/-) or TRAIL-R1 and TRAIL-R2-deficient (DKO) HCT116 cells within the TRAIL-R1 and TRAIL-R2 loci targeted by the corresponding TALENs are shown. Figures on the upper left inside correspond to the number of sequences read from each sample. Figures shown on the upper right inside correspond to percentage of sequence displaying mutations within the locus. Deletions are represented in white bars above the sequence shown on the baseline. Insertions are shown in black and substitutions in green. Note that the DKO was generated from the TRAIL-R1 population (DR4 -/-). **C.** Isogenic HCT116 cells were stimulated with increasing concentrations of Fc-FasL (FasL) for 16 hours and cell viability was determined by methylene blue staining. Data represents mean ± SD of three independent experiments.

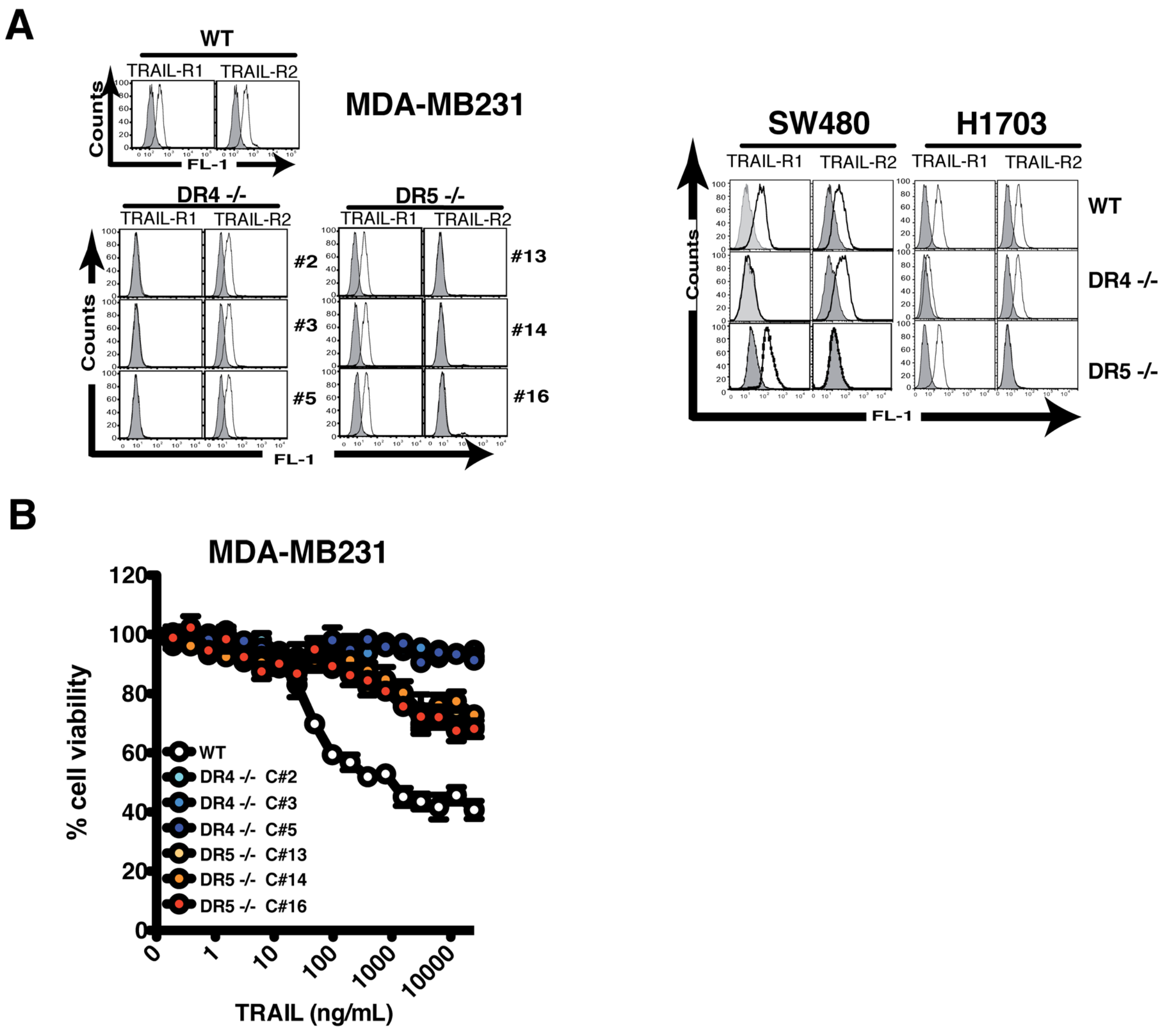

Supplementary Figure 2: Generation and characterization of TRAIL-R1- and TRAIL-R2-deficient isogenic MDA-MB23, SW480 and H1703 cells. A. Parental (WT), TRAIL-R1-deficient (DR4 -/-) and TRAIL-R2-deficient (DR5 -/-) MDA-MB-231, SW480 and H1703 populations or clones were assessed by flow cytometry for TRAIL-R1 or TRAIL-R2 expression, as indicated. **B.** Cell viability of parental (WT), DR4 -/- clones (#2; #3 and #5) or DR5 -/- clones (#13, #14 and #16) MDA-MB-231 cells to increasing concentrations of recombinant TRAIL (His-TRAIL), assessed by methylene blue 16 h after stimulation.

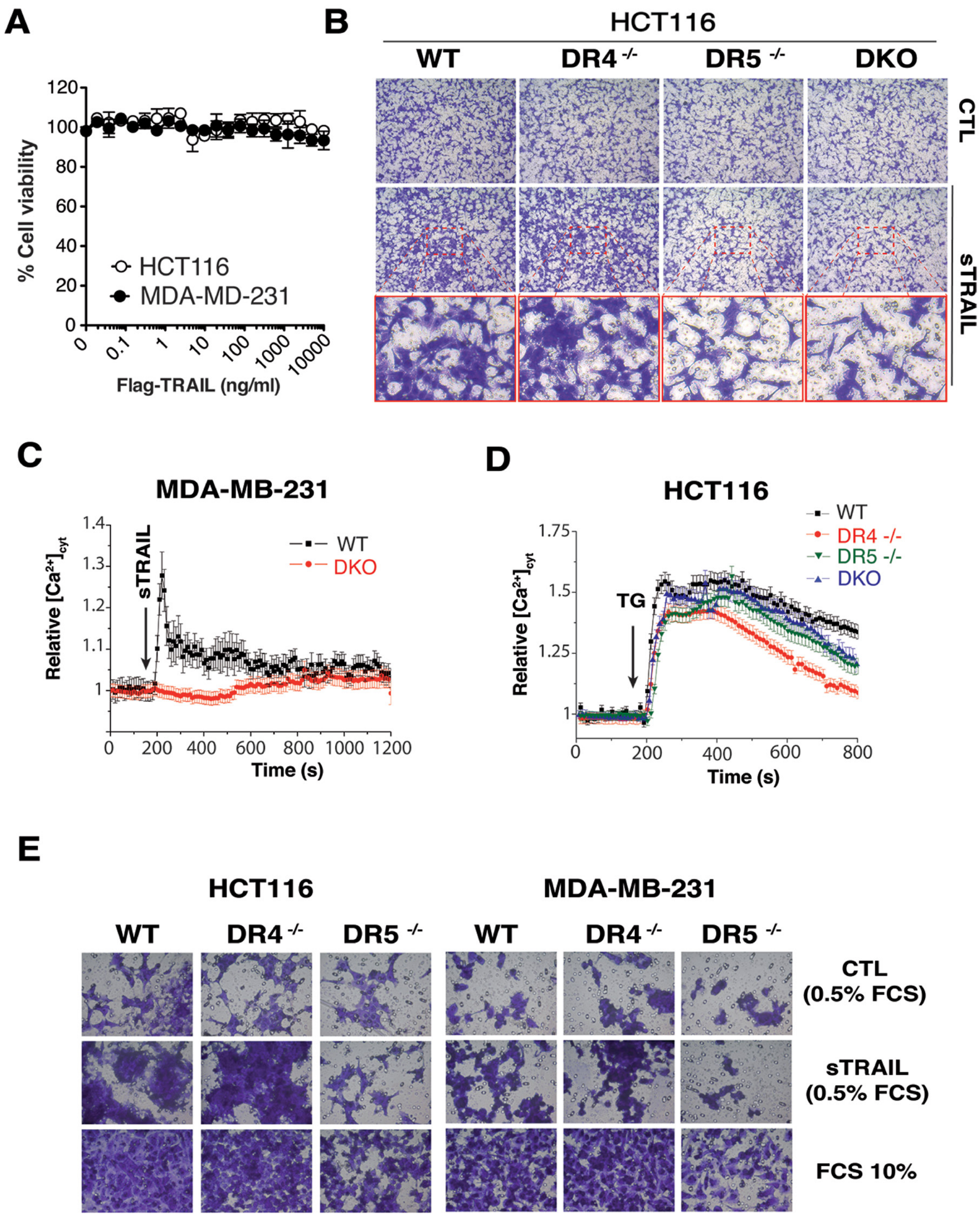

Supplementary Figure 3: TRAIL-R2, but not TRAIL-R1 induces cell motility in HCT116 cells after sTRAIL stimulation. **A.** Parental HCT116 and MDA-MB-231 cells exposed to increasing concentrations of Flag-TRAIL for 16 hours. Cell viability was assessed by methylen blue staining. **B.** Parental (WT) and TRAIL-R1- (DR4-/-), TRAIL-R2- (DR5-/-) and/or TRAIL-R1 and TRAIL-R2-deficient (DKO) HCT116 cells were starved overnight, seeded in the presence of low serum (0.5%) with or without Flag-TRAIL (100 ng/ml) for 24 h in a boyden chamber and migration was assessed by staining with Giemsa. A representative image of three independent experiments is shown. **C-D.** $[Ca^{2+}]_{CYT}$ was assessed in FuraPE3-AM (1 µM)-loaded on indicated cells. Ratio values (R=F340/F380) were normalized to pre-stimulated values (R0). Data represent means ± the SD of 3 independent experiments (> 60 cells). Shown are time course of calcium responses to sTRAIL (100 ng/ml) in parental and DKO MDA-MB-231 cells (C) and to 2 µM thapsigargin (TG) in parental, DR4 -/-, DR5 -/- or DKO HCT116 cells (D). **E.** Parental (WT) and TRAIL-R1- (DR4-/-) or TRAIL-R2- (DR5-/-) HCT116 or MDA-MB-231 cells were starved overnight, seeded for 24 h in the presence of low (0.5%) or high serum (10%) with or without Flag-TRAIL (100 ng/ml) in a boyden chamber and migration was assessed by staining with Giemsa. A representative image of three independent experiments is shown.

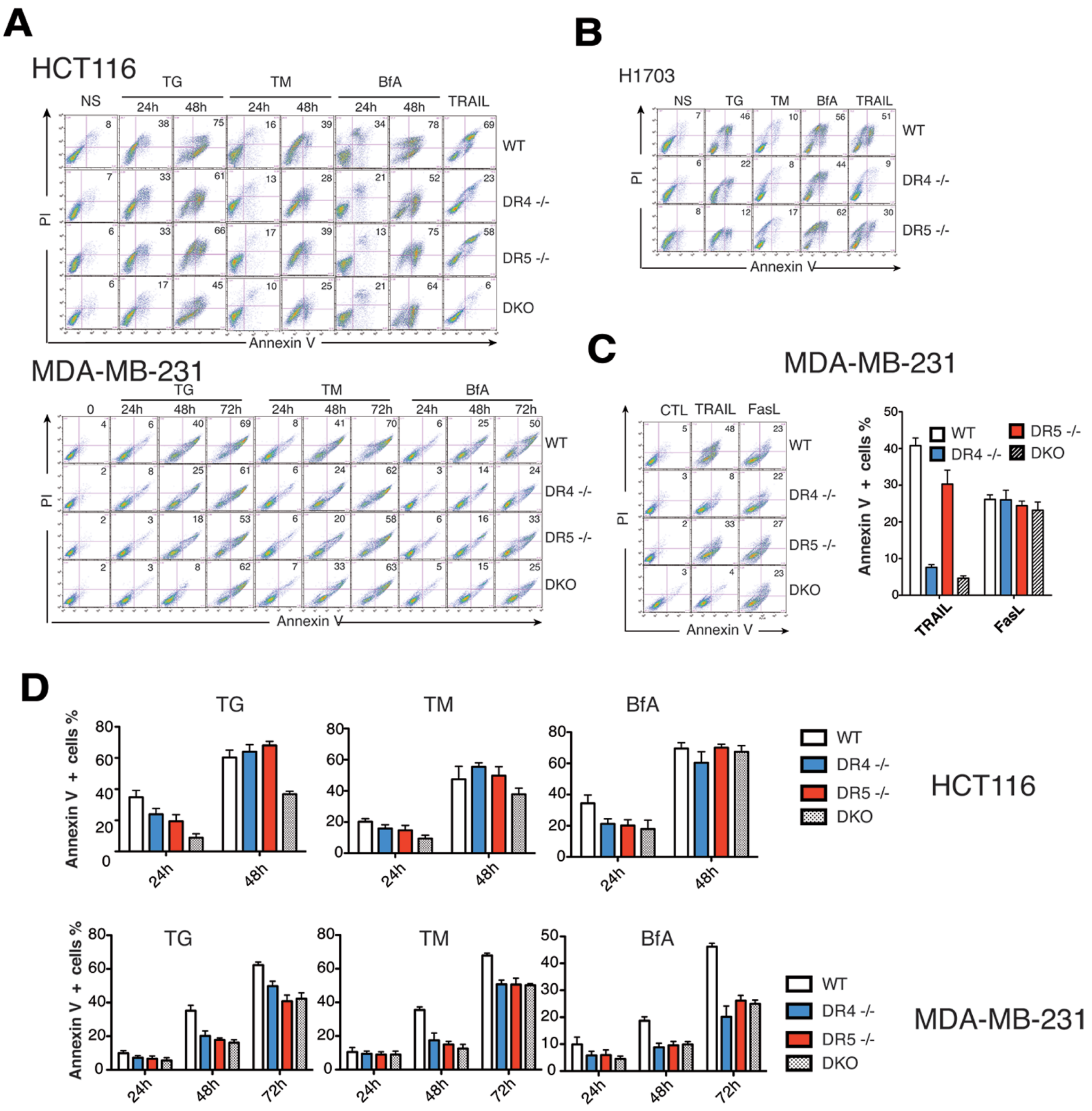

Supplementary Figure 4: ER stress-mediated apoptosis in HCT116, MDA-MB23 and H1703 TRAIL-Receptor-deficient isogenic cell derivatives. A. Parental (WT), TRAIL-R1-deficient (DR4 -/-), TRAIL-R2-deficient (DR5 -/-) and TRAIL-R1/R2-deficient (DKO) HCT116 or MDA-MB-231 cells were stimulated with 1 µg/ml brefeldin A (BfA), 1 µg/ml tunicamycin (TM) or 100 nM thapsigargin (TG) for the indicated period of time and analyzed for annexin V staining by flow cytometry. Annexin V/PI % positive cells are shown in the upper right corner of each representative histogram. **B.** H1703 parental or TRAIL-receptor-deficient cells were stimulated as above and analyzed for apoptosis. **C.** Indicated MDA-MB-231 isogenic cells were stimulated with 1 µg/ml His-TRAIL (TRAIL) or 1 µg/ml Fc-FasL (FasL) and apoptosis was analyzed as above. Right panel: Annexin V staining quantification of at least 3 independent experiments. **D.** Quantification of apoptosis in HCT116 and MDA-MB-231 isogenic TRAIL receptor-deficient cells induced by 1 µg/ml brefeldin A (BfA), 1 µg/ml tunicamycin (TM) or 100 nM thapsigargin (TG) for the indicated period of time. Data represent the mean ± SD of at least three different experiments. (***P<0.05 respective to parental cells, one-way ANOVA).
